# Very high-power short-duration using 70W and a flexible tip ablation catheter for pulmonary vein isolation: the POWER PULSE randomized controlled trial

**DOI:** 10.1093/europace/euaf105

**Published:** 2025-07-21

**Authors:** Miruna A Popa, Hannah Krafft, Fabian Bahlke, Florian Englert, Sarah Lengauer, Marta Telishevska, Nico Erhard, Madeleine Tydecks, Martin Hadamitzky, Felix Bourier, Tilko Reents, Elisabeth Klupp, Carsten Lennerz, Gabriele Hessling, Isabel Deisenhofer, Marc Kottmaier

**Affiliations:** Department of Electrophysiology, German Heart Center Munich, Technical University of Munich, Lazarettstraße 36, Munich 80636, Germany; Munich Arrhythmia Research and Study Center (MARS), Technical University of Munich, Lazarettstraße 36, Munich 80636, Germany; Department of Electrophysiology, German Heart Center Munich, Technical University of Munich, Lazarettstraße 36, Munich 80636, Germany; Munich Arrhythmia Research and Study Center (MARS), Technical University of Munich, Lazarettstraße 36, Munich 80636, Germany; Department of Electrophysiology, German Heart Center Munich, Technical University of Munich, Lazarettstraße 36, Munich 80636, Germany; Munich Arrhythmia Research and Study Center (MARS), Technical University of Munich, Lazarettstraße 36, Munich 80636, Germany; Department of Electrophysiology, German Heart Center Munich, Technical University of Munich, Lazarettstraße 36, Munich 80636, Germany; Munich Arrhythmia Research and Study Center (MARS), Technical University of Munich, Lazarettstraße 36, Munich 80636, Germany; Department of Electrophysiology, German Heart Center Munich, Technical University of Munich, Lazarettstraße 36, Munich 80636, Germany; Munich Arrhythmia Research and Study Center (MARS), Technical University of Munich, Lazarettstraße 36, Munich 80636, Germany; Department of Electrophysiology, German Heart Center Munich, Technical University of Munich, Lazarettstraße 36, Munich 80636, Germany; Munich Arrhythmia Research and Study Center (MARS), Technical University of Munich, Lazarettstraße 36, Munich 80636, Germany; Department of Electrophysiology, German Heart Center Munich, Technical University of Munich, Lazarettstraße 36, Munich 80636, Germany; Munich Arrhythmia Research and Study Center (MARS), Technical University of Munich, Lazarettstraße 36, Munich 80636, Germany; Department of Electrophysiology, German Heart Center Munich, Technical University of Munich, Lazarettstraße 36, Munich 80636, Germany; Munich Arrhythmia Research and Study Center (MARS), Technical University of Munich, Lazarettstraße 36, Munich 80636, Germany; Institute for Radiology and Nuclear Medicine, German Heart Center Munich, Technical University of Munich, Lazarettstraße 36, Munich 80636, Germany; Department of Electrophysiology, German Heart Center Munich, Technical University of Munich, Lazarettstraße 36, Munich 80636, Germany; Munich Arrhythmia Research and Study Center (MARS), Technical University of Munich, Lazarettstraße 36, Munich 80636, Germany; Department of Electrophysiology, German Heart Center Munich, Technical University of Munich, Lazarettstraße 36, Munich 80636, Germany; Munich Arrhythmia Research and Study Center (MARS), Technical University of Munich, Lazarettstraße 36, Munich 80636, Germany; Department of Diagnostic and Interventional Neuroradiology, Klinikum Rechts der Isar, Technical University of Munich, Ismaninger Str. 22, Munich 81675, Germany; Department of Electrophysiology, German Heart Center Munich, Technical University of Munich, Lazarettstraße 36, Munich 80636, Germany; Munich Arrhythmia Research and Study Center (MARS), Technical University of Munich, Lazarettstraße 36, Munich 80636, Germany; Department of Electrophysiology, German Heart Center Munich, Technical University of Munich, Lazarettstraße 36, Munich 80636, Germany; Munich Arrhythmia Research and Study Center (MARS), Technical University of Munich, Lazarettstraße 36, Munich 80636, Germany; Department of Electrophysiology, German Heart Center Munich, Technical University of Munich, Lazarettstraße 36, Munich 80636, Germany; Munich Arrhythmia Research and Study Center (MARS), Technical University of Munich, Lazarettstraße 36, Munich 80636, Germany; Department of Electrophysiology, German Heart Center Munich, Technical University of Munich, Lazarettstraße 36, Munich 80636, Germany; Munich Arrhythmia Research and Study Center (MARS), Technical University of Munich, Lazarettstraße 36, Munich 80636, Germany

**Keywords:** Atrial fibrillation, Radiofrequency ablation, High-power short-duration

## Abstract

**Aims:**

Very high-power short-duration (vHPSD) was developed to optimize radiofrequency ablation for atrial fibrillation (AF). However, data on vHPSD ≥ 70W remains limited. We investigated acute efficacy, safety and long-term rhythm outcomes of vHPSD-70W in a randomized controlled trial.

**Methods and results:**

A total of *n* = 200 patients with paroxysmal AF were randomly assigned 1:1 to receive pulmonary vein isolation (PVI) using vHPSD (70 W/5–7 s) or standard (30–40W, 20–40 s) ablation with a flexible, enhanced-irrigation tip catheter. Primary endpoint was the number of reconnected pulmonary veins (rPV) after adenosine testing. Secondary endpoints included first-pass isolation (FPI), silent cerebral lesions (SCLs) and rhythm outcomes on 12-month follow-up. Mean number of rPVs was 0.6 ± 0.8 vs. 0.8 ± 0.9 (*P* = 0.145) with vHPSD-70W vs. standard ablation. Bilateral FPI was 42.7% vs. 30.2% (*P* = 0.072), while FPI of left PVs was higher with vHPSD-70W (63.5% vs. 49.0%, *P* = 0.042). Procedure (107.7 ± 34.2 vs. 131.3 ± 42.2 min) and radiofrequency (15.1 ± 6.7 vs. 41.8 ± 18.3 min) duration were significantly lower with vHPSD-70W (*P* < 0.001). Silent cerebral lesions occurred in 1/25 (4.0%) vs. 3/22 (13.6%, *P* = 0.328). On 12-month follow-up, freedom from any atrial arrhythmia (76.0% vs. 66.7%, *P* = 0.171) was similar, while vHPSD-70W showed a lower incidence of atrial tachycardia (AT) recurrence (1.0% vs. 10.4%, *P* = 0.005).

**Conclusion:**

Very high-power short-duration with 70 W/5–7 s was non-superior to standard ablation regarding acute PV reconnection and 12-month freedom from any atrial arrhythmia. However, vHPSD-70W achieved a higher FPI rate of left PVs with a shorter procedure duration and a comparable safety profile. AT recurrence was significantly less common with vHPSD-70W.

## Graphical Abstract

**Graphical Abstract:**
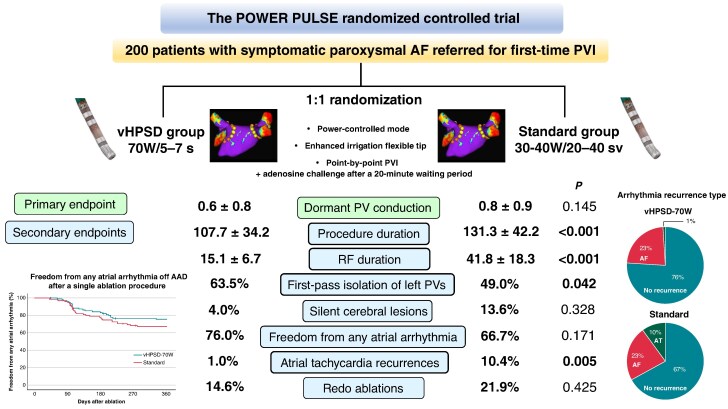

What’s new?

- Very high-power short-duration with 70W/5–7 s (vHPSD-70W) is non-superior to standard ablation (30–40W, 20–40 s) in terms of dormant pulmonary vein conduction and 12-month freedom from any atrial arrhythmia
- vHPSD-70W increases the first-pass isolation rate of left-sided PVs and reduces procedure and radiofrequency duration compared to standard ablation
- vHPSD-70W shows a similar safety profile as standard ablation and is not associated with an increased incidence of silent cerebral lesions
- vHPSD-70W is associated with a significant reduction in atrial tachycardia recurrences

## Introduction

Pulmonary vein isolation (PVI) represents the cornerstone of atrial fibrillation (AF) ablation and has advanced to a first-line treatment strategy for AF.^1,2^ As AF prevalence is continuously increasing due to an ageing population, novel technologies are emerging to improve efficacy and safety of catheter ablation.

Standard radiofrequency ablation employs thermal energy at low power (20–40 W) for long duration (20–40 s) with the aim of creating contiguous, transmural and durable lesions.^3,4^ Despite technological advancements, it remains limited by non-uniform, partly reversible lesions favouring PV reconnections and by the risk of collateral tissue damage. In recent years, an optimized ablation protocol has been introduced which employs higher power (>40 W) for shorter duration (<20 s) to improve lesion quality and safety profile by modifying the relationship between resistive and conductive heating phase.^5^ Pre-clinical data from animal studies showed that high-power short-duration (HPSD) lesions are shallower and wider than with standard ablation and are expected to be more durable due to less tissue oedema.^5–7^

While retrospective studies have consistently demonstrated a shorter procedure duration with HPSD,^4,7–11^ the benefit of this ablation protocol for procedure efficacy, long-term rhythm outcomes and safety remains less well established. Despite a small number of randomized controlled trials (RCTs), a definite appraisal of HPSD ablation remains hampered by a wide range of HPSD definitions (40–90 W), heterogeneous ablation protocols, various ablation catheters and small study populations used throughout these studies.^12–18^ Data from RCTs on very high-power short-duration (vHPSD) ≥ 70 W remain scarce. In two retrospective studies we found a shorter procedure duration, a similar safety profile and superior 12-month rhythm outcomes for vHPSD with 60–70 W as compared to standard ablation.^10,11^

This randomized-controlled study was designed to investigate whether vHPSD ablation using 70 W/5–7 s is superior to standard ablation in terms of acute PVI efficacy. It was based on the hypothesis that vHPSD improves lesion quality by creating transmural, irreversible lesions, which result in a lower rate of dormant PV conduction on adenosine challenge. Secondary endpoints included safety and long-term outcome.

## Methods

### Trial design

**Figure 1:**
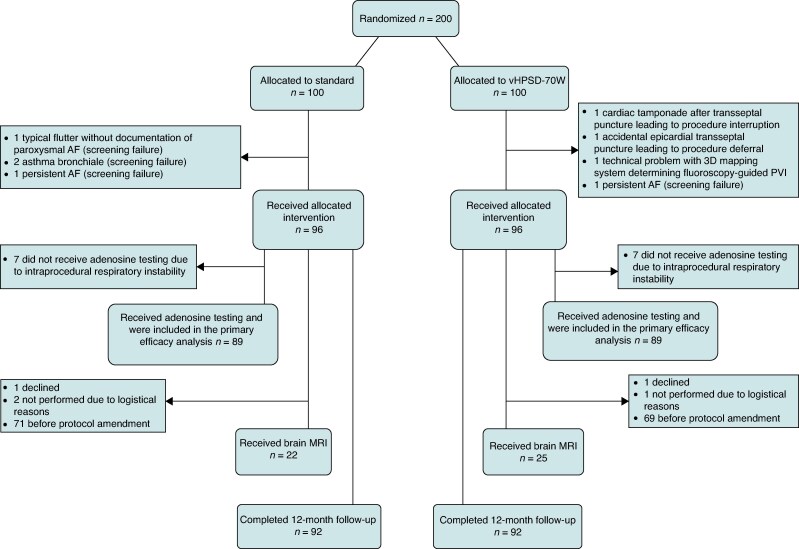
Study flow diagram. AF, atrial fibrillation, MRI, magnetic resonance imaging, PVI, pulmonary vein isolation, vHPSD, very high-power short-duration.

The POWER PULSE study was a prospective, single-center, two-arm, parallel-group, randomized controlled trial conducted at the German Heart Centre Munich, Germany. A total of 200 participants were randomly assigned either to receive standard ablation or vHPSD ablation. Randomization was performed using permuted-block randomization with a block size of 4 and an allocation ratio of 1:1. Allocation concealment was ensured by using sequentially numbered, opaque, sealed envelopes and by blinding the study group to the randomization method and block size. Randomization and patient enrolment were coordinated and supervised by the principal investigator of the study. A data safety monitoring board of independent researchers was established. The study flow diagram is depicted in *Figure 1*.

The study was conducted according to the principles of the declaration of Helsinki and approved by the local ethics committee (approval #210/18 S-AS). All participants provided written informed consent. The trial was registered at ClinicalTrials.gov (NCT05959798). A study amendment was approved as of July 2020 which allowed brain magnetic resonance imaging (MRI) to detect silent cerebral lesions (SCLs) in consecutive patients within 72 h after the procedure. The study amendment was determined by contemporary interest in this outcome and not by any safety concern arising from the current study.

### Participants

Recruitment was performed between January 2019 and June 2022. Consecutive patients aged between 18 and 80 years with documented, symptomatic, drug-refractory paroxysmal AF referred for first-time PVI were eligible for study participation. A detailed list of inclusion and exclusion criteria is found in the Supplementary material. After randomization, 4 participants in each group were excluded prior to receiving the allocated intervention, leaving *n* = 96 patients in the standard group and *n* = 96 patients in the vHPSD-70W group for the primary analysis (*Figure 1*).

### Study endpoints

Primary endpoint was the number of reconnected PVs on adenosine testing after a 20-min waiting period. Secondary efficacy endpoints included procedure duration, first-pass isolation (FPI) and rhythm outcomes at 12-month follow-up. Secondary safety endpoints included the incidence of steam pops, the incidence of SCLs and procedure-related complications. A detailed list of pre-defined study endpoints is found in the Supplementary material.

### Ablation protocol

Left atrial thrombus was excluded ≤ 48 h before ablation using contrast-enhanced cardiac computed tomography or transoesophageal echocardiography. A three-dimensional segmentation of LA anatomy and oesophagus position relative to the LA posterior wall was reviewed during the procedure. Antiarrhythmic drugs were discontinued ≥3 half-lives prior to the procedure and were not re-started afterwards.

The ablation procedure was performed on uninterrupted oral anticoagulation and under deep sedation using midazolam, propofol and fentanyl. After gaining venous femoral access, fluoroscopy-guided single transseptal puncture was performed using the Agilis™ steerable sheath (Abbott, Plymouth, MN) and double access to the LA was obtained. Unfractionated heparin was administered after transseptal puncture to achieve an activated clotting time ≥ 300 s throughout the entire procedure. High-density electroanatomical mapping was performed using EnSite™ Precision (Abbott, Plymouth, MN) and a circular mapping catheter (Orbiter™ PV, Boston Scientific, Marlborough, MA). Wide antral circumferential PVI was performed using point-by-point-lesions with the 4 mm enhanced irrigated-tip catheter FlexAbility™ SE and the Ampere® RF generator (Abbott, Plymouth, MN).

Participants were randomly assigned to either receive standard ablation with 30–40 W (30–40 s on the anterior wall and 20 s on the posterior wall) or vHPSD ablation with 70 W (7 s on the anterior wall and 5 s on the posterior wall). A power-controlled mode using an automated control for RF delivery duration and an automatic temperature cut-off set at 42°C were used in both study groups. Irrigation was set to 17 mL/min (standard group) and to 20 mL/min (vHPSD-70W group). The targeted baseline impedance was 110–140 Ω in both groups. If impedance was >140 Ω, repositioning of the neutral electrode or placement of a second neutral electrode were performed. Real-time automated display of RF applications was performed using the AutoMark module (tag size 3 mm/minimum time 10 s for standard ablation, tag size 4 mm/minimum time 2 s for HPSD ablation). An inter-lesion distance of ≤6 mm (standard) and 5–6 mm (vHPSD-70W) was targeted. Lesion overlapping was avoided with vHPSD for safety reasons due to previously reported higher lesion diameter.^5,6^ Oesophageal temperature probes were not employed in this study. After completing the PVI circle around ipsilateral PVs, entrance block was verified by placing the circular mapping catheter in each PV ostium. In case FPI of ipsilateral PV pairs was not achieved, additional RF lesions were applied at the site of earliest activation. A 20-min waiting period was implemented prior to adenosine challenge for each PV pair, as previously described.^19^ Spontaneous reconnections occurring after the waiting period were addressed by touch-up lesions prior to adenosine challenge. Dormant PV conduction was then assessed by administering at least 12 mg adenosine intravenously in a dose which would induce transient AV block. Each PV was tested separately and sequentially by placing the circular mapping catheter in the PV ostium and administering the adenosine bolus thereafter. In case of acute PV reconnection, additional ablation lesions were applied at the site of earliest activation until dormant PV conduction on repeat adenosine challenge was no longer present.

Ablation of the cavotricuspid isthmus was performed in case of intraprocedural occurrence of typical flutter using moderate power settings (40–45 W/30 s) in both groups.

### Outcomes

Transthoracic echocardiography to assess pericardial effusion was routinely performed at the end of each procedure, the following morning and at any time in case of haemodynamic deterioration. The vascular access site was clinically inspected the day after the procedure and complemented by duplex sonography in case of clinical signs suggestive of vascular complications. All patients received medication with pantoprazole 40 mg twice daily for 4 weeks following PVI.

Native brain MRI was performed in *n* = 47 patients at 1.5 Tesla 24–72 h after ablation using the following sequences: (i) T2-w TSE axial (5 mm), (ii) FLAIR axial (5 mm), (iii) DWI-EPI (5 mm) *b*-values 0 and 1000 s/mm² with calculated ADC values. Silent cerebral lesions were defined as DWI hyperintense, ADC reduced and FLAIR positive, as previously described.^20^ Image analysis was performed by two independent neuroradiologists who were blinded to the group allocation. Systematic neurological evaluation using the National Institutes of Health Stroke Scale (NIHSS) before ablation and at discharge was performed in all patients receiving brain MRIs.

All participants received a clinical assessment and 7-day-Holter ECGs during each follow-up visit scheduled at 3-, 6-, and 12-months in our outpatient clinic. Arrhythmia recurrence was defined as any atrial arrhythmia ≥30 s documented after a blanking period of 6 weeks. Arrhythmia assessment was based on 7-day-Holter ECGs and on any other ECG documentation presented during follow-up. Data from any redo ablation procedure due to atrial arrhythmia recurrence was also assessed.

### Statistical analysis

The study was designed to investigate the superiority of vHPSD-70W over standard ablation regarding dormant PV conduction. The required sample size was calculated by estimating a mean PV reconnection of 1.0 ± 1.0 in the standard group, based on the results of the ADVICE trial, which reported that 21% of PVs show dormant conduction on adenosine challenge.^19^ Assuming a mean reduction of PV reconnection in the vHPSD group by 40%,^5,10^ a statistical power of 0.8, a Type I error of 0.05 and a drop-out rate of 2%, a sample size of 200 participants was calculated.

Continuous variables are expressed as mean ± standard deviation or median and interquartile range (25th and 75th percentile) and compared by two-sample *t*-tests. Categorical variables are presented as frequencies or percentages and compared by χ2 test (or Fisher’s exact test in case of small proportions). Time to first AF/AT recurrence was plotted using the Kaplan–Meier product limit method and compared by the log-rank test. Statistical tests and confidence intervals with two-tailed *P* < 0.05 were considered statistically significant. Statistical analysis was performed using SPSS software version 28.0 (IBM Inc., Armonk, NY).

## Results

### Baseline and procedural characteristics

A total of 200 participants underwent randomization, of which *n* = 192 received the allocated intervention (*n* = 96 vHPSD-70W, *n* = 96 standard approach) and were included in the analysis (*Figure 1*). Baseline and procedural characteristics are summarized in *Table 1* and were well-balanced between groups, apart from a higher diagnosis-to-ablation time found with vHPSD-70W.

**Table 1:** Baseline and procedural characteristics

	vHPSD-70W (*n* = 96)	Standard (*n* = 96)	*P*-value
Baseline characteristics			
Age, years	63.6 ± 9.8	62.0 ± 10.4	0.265
Male, *n* (%)	54 (56.3)	63 (65.6)	0.183
Body mass index, kg/m^2^	26.6 ± 4.4	27.0 ± 4.6	0.544
Hypertension, *n* (%)	52 (54.2)	41 (42.7)	0.112
Diabetes, *n* (%)	6 (6.3)	7 (7.3)	0.774
Vascular disease, *n* (%)	23 (24.0)	20 (20.8)	0.604
Previous stroke or transient ischaemic attack, *n* (%)	6 (6.3)	4 (4.2)	0.516
Heart failure, *n* (%)	6 (6.3)	13 (13.5)	0.091
CHA_2_DS_2_VASC Score	2 (1; 3)	2 (1; 3)	0.435
Median diagnosis-to-ablation time, months	16 (6; 38)	12 (5; 36)	0.047
Median duration of AF episodes, h	8 (2; 24)	4 (2; 24)	0.975
Left atrial surface, cm^2^	22.3 ± 4.4	22.7 ± 4.6	0.653
Left atrial diameter, mm	41.9 ± 8.8	39.3 ± 7.1	0.205
Implanted rhythm monitoring device, *n* (%)	2 (2.1)	3 (3.1)	1.000
Procedural data			
Pulmonary vein isolation, *n* (%)	96 (100)	96 (100)	1.000
Additional ablation, *n* (%)	7 (7.3)	7 (7.3)	1.000
Cavotricuspid isthmus line, *n* (%)	4 (4.2)	5 (5.2)	1.000
Slow pathway ablation, *n* (%)	1 (1.0)	2 (2.1)	1.000
Focal atrial tachycardia, *n* (%)	1 (1.0)	1 (1.0)	1.000
Roof line, *n* (%)	1 (1.0)	0 (0.0)	1.000
Mean temperature, °C	31.6 ± 3.1	31.4 ± 2.4	0.569
Mean power, W	55.7 ± 5.5	33.9 ± 6.9	<0.001
Mean radiofrequency duration per lesion, s	6.5 ± 0.4	30.3 ± 9.3	<0.001
Procedure duration, min	107.7 ± 34.2	131.3 ± 42.2	<0.001
Radiofrequency duration, min	15.1 ± 6.7	41.8 ± 18.3	<0.001
Fluoroscopy duration, min	8.0 ± 11.4	6.4 ± 3.2	0.184
Adenosine testing, *n* (%)	89 (92.7)	89 (92.7)	1.000

AF, atrial fibrillation.

Pulmonary vein isolation was achieved in 100% of participants in both groups (*Table 1*). Additional ablation was performed in 7/96 (7.3%) in both vHPSD-70W and standard groups and was determined by intraprocedural occurrence of typical flutter (4 vs. 5), slow-fast AV-nodal reentry tachycardia (1 vs. 2), focal atrial tachycardia (1 vs. 1), and roof-dependent atrial tachycardia (AT) (1 vs. 0).

### Acute efficacy outcomes

Adenosine testing was performed in 89/96 (92.7%) of participants in both groups. The number of reconnected PVs after a 20-min waiting period and adenosine testing was slightly lower with vHPSD-70W than with standard ablation, while it did not reach statistical significance (0.6 ± 0.8 vs. 0.8 ± 0.9, *P* = 0.145, *Table 2*). Acute reconnection of at least one PV after adenosine testing was observed in 39/89 (43.8%) vs. 50/89 (56.2%) patients (*P* = 0.099). First-pass isolation of left-sided PVs was significantly higher with vHPSD-70W (61/96 [63.5%]) as compared to standard ablation (47/96 [49.0%], *P* = 0.042) and there was a trend towards a higher FPI of both PV pairs with vHPSD-70W (41/96 [42.7%] vs. 29/96 [30.2%], *P* = 0.072).

**Table 2:** Intraprocedural efficacy endpoints

	vHPSD-70W (*n* = 96)	Standard (*n* = 96)	*P*-value
Primary efficacy endpoint			
Number of reconnected PVs	0.6 ± 0.8	0.8 ± 0.9	0.145
Secondary efficacy endpoints			
Any PV reconnection after adenosine	39/89 (43.8)	50/89 (56.2)	0.099
≥ 2 reconnected PVs after adenosine	13/89 (14.6)	16/89 (18.0)	0.543
≥ 3 reconnected PVs after adenosine	2/89 (2.3)	4/89 (4.5)	0.682
First-Pass Isolation of both PV pairs	41/96 (42.7)	29/96 (30.2)	0.072
First-Pass Isolation of Left PVs	61/96 (63.5)	47/96 (49.0)	0.042
First-Pass Isolation of Right PVs	58/96 (60.5)	49/96 (50.0)	0.191
Effectiveness of both PV pairs	21/89 (23.6)	15/89 (16.9)	0.263
Effectiveness of Left PVs	42/89 (47.2)	35/89 (39.3)	0.290
Effectiveness of Right PVs	42/89 (47.2)	35/89 (39.3)	0.290

PV, pulmonary vein. Effectiveness measures are defined as both ipsilateral first-pass isolation and absence of acute reconnection upon adenosine testing.

Procedure duration (107.7 ± 34.2 vs. 131.3 ± 42.2 min) and radiofrequency duration (15.1 ± 6.7 vs. 41.8 ± 18.3 min) were significantly lower with vHPSD-70W as compared to standard ablation (both *P* < 0.001). Fluoroscopy duration was similar between the groups (8.0 ± 11.4 vs. 6.4 ± 3.2 min, *P* = 0.184).

### Safety outcomes

Safety endpoints are listed in *Table 3*. No statistically significant differences in the rate of steam pops (5/96 [5.2%] vs. 0/96 [0.0%], *P* = 0.059), SCLs (1/25 [4.0%] vs. 3/22 [13.6%], *P* = 0.328) and transient ischaemic attacks (1/96 [1.0%] vs. 0/96 [0.0%], *P* = 1.000) were observed between vHPSD-70W and standard approach. No patient in the vHPSD-70W group presented any oesophageal symptoms, while two patients in the standard group required endoscopy due to symptoms occurring after 1 and 4 days, respectively. In the latter patient, an oesophageal thermal lesion was confirmed, which resolved after treatment with proton pump inhibitors. No stroke, cardiac arrest or death occurred in any of the groups. Emergent re-admission due to recurrence of any atrial arrhythmia occurred in 2/96 (2.1%) vs. 8/96 (8.3%) after vHPSD-70W vs. standard ablation (*P* = 0.051).

**Table 3:** Safety endpoints

	vHPSD-70W (*n* = 96)	Standard (*n* = 96)	*P*-value
Steam pop, n (%)	5/96 (5.2)	0/96 (0.0)	0.059
Silent cerebral lesions, *n* (%)	1/25 (4.0)	3/22 (13.6)	0.328
Vascular access complication, *n* (%)	7/96 (7.3)	2/96 (2.1)	0.169
Transient ischaemic attack, *n* (%)	1/96 (1.0)	0/96 (0.0)	1.000
Stroke, *n* (%)	0/96 (0.0)	0/96 (0.0)	1.000
Neurological deficit assessed by NIHSS, *n* (%)	0/25 (0.0)	0/22 (0.0)	1.000
Cardiac tamponade with pericardiocentesis, *n* (%)	0/96 (0.0)	0/96 (0.0)	1.000
Emergent readmission due to arrythmia recurrence, *n* (%)	2/96 (2.1)	8/96 (8.3)	0.051
Pacemaker implantation within 3 months, *n* (%)	2/96 (2.1)	2/96 (2.1)	1.000
Syncope related to recurrence, *n* (%)	0/96 (0.0)	3/96 (3.1)	0.246
Oesophageal symptoms requiring endoscopy, *n* (%)	0/96 (0.0)	2/96 (2.1)	0.497
Atrio-oesophageal fistula, *n* (%)	0/96 (0.0)	0/96 (0.0)	1.000
Uncomplicated infection, *n* (%)	3/96 (3.1)	1/96 (1.0)	0.621
Cardiac arrest, *n* (%)	0/96 (0.0)	0/96 (0.0)	1.000
Death, *n* (%)	0/96 (0.0)	0/96 (0.0)	1.000

NIHSS, National Institutes of Health Stroke Scale.

### Long-term rhythm outcomes

**Figure 2:**
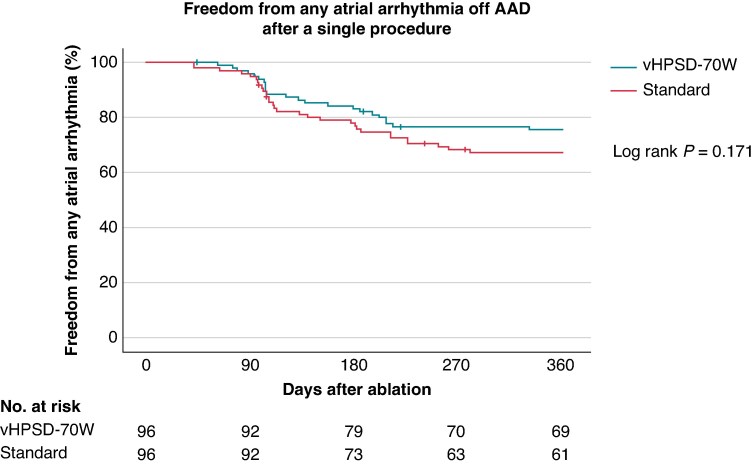
Kaplan–Meier analysis of freedom from any atrial arrhythmia after a blanking period of 6 weeks. AAD, antiarrhythmic drugs, vHPSD, very high-power short-duration.

**Figure 3:**
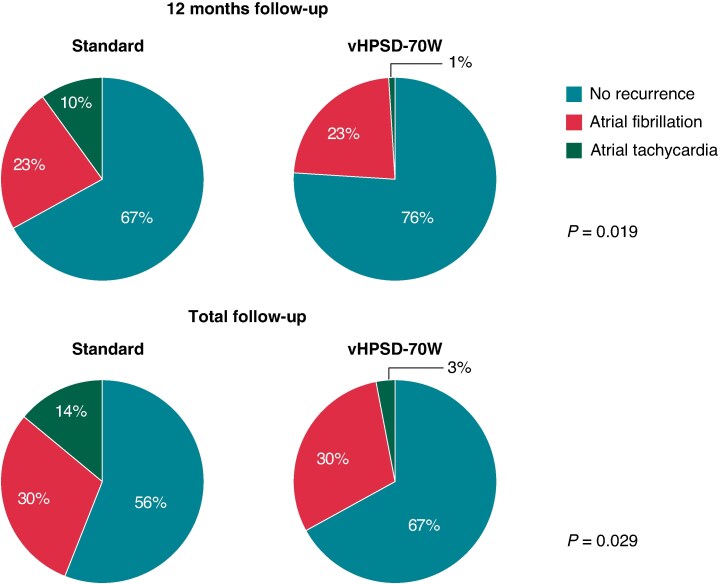
Type of arrhythmia recurrence at 12 months and by the end of total follow-up. vHPSD, very high-power short-duration.

Follow-up was completed by 92/96 (95.8%) patients in both groups. The 12-month Kaplan–Meier estimates of freedom from any atrial arrhythmia after a single procedure off antiarrhythmic drugs were 76.0% vs. 66.7% (Log Rank *P* = 0.171, *Figure 2*). Atrial fibrillation recurrence occurred in 22/96 (22.9%) patients in both groups, while vHPSD-70W was associated with a significantly lower rate of AT recurrence (1/96 [1.0%] vs. 10/96 [10.4%], *P* = 0.005, *Figure 3*, *Table 4*). A total of 5/10 (50.0%) patients with AT recurrence in the standard group required emergent re-admission due to poorly tolerated AT. vHPSD-70W was associated with a statistically not significant lower rate of redo ablations within 12 months (14/96 [14.6%] vs. 21/96 [21.9%], *P* = 0.191).

**Table 4:** Clinical rhythm outcomes

	vHPSD-70W (*n* = 96)	Standard (*n* = 96)	*P*-value
Early recurrence of any atrial arrhythmia, *n* (%)	18/96 (18.8)	21 (21.9)	0.591
Clinical rhythm outcomes at 12 months			
Recurrence of any atrial arrhythmia, *n* (%)	23/96 (24.0)	31/96 (32.3)	0.199
Recurrence of atrial fibrillation, *n* (%)	22/96 (22.9)	22/96 (22.9)	1.000
Recurrence of atrial tachycardia, *n* (%)	1/96 (1.0)	10/96 (10.4)	0.005
Emergent re-admission due to AT, *n* (%)	0/96 (0.0)	5/96 (5.2)	0.059
Redo ablation, *n* (%)	14/96 (14.6)	21/96 (21.9)	0.191
Clinical rhythm outcomes by the end of follow-up			
Recurrence of any atrial arrhythmia, *n* (%)	32/96 (33.3)	42/96 (43.8)	0.138
Recurrence of atrial fibrillation, *n* (%)	29/96 (30.2)	29/96 (30.2)	1.000
Recurrence of atrial tachycardia, *n* (%)	3/96 (3.1)	13/96 (13.5)	0.009
Emergent re-admission due to AT, *n* (%)	0/96 (0.0)	6/96 (6.3)	0.029
Redo ablation, *n* (%)	25/96 (26.0)	30/96 (31.3)	0.425
Chronic reconnection on redo ablation, *n* (%)	23/25 (92.0)	28/30 (93.3)	1.000
Progression to persistent AF, *n* (%)	4/96 (4.2)	4/96 (4.2)	1.000

After a median follow-up duration of 437 days [IQR 370; 827] in the vHPSD-70W group and 414 days [IQR 367; 668] in the standard group (*P* = 0.187), redo ablation was performed in 25 and 30 patients, respectively (*P* = 0.425). Procedural details of redo ablations are summarized in Supplementary material online, *Table S1*. The mean number of reconnected PVs and the rate of chronic PV reconnections were similar between groups. On extended follow-up, AT occurred in 3/96 (3.1%) in the vHPSD-70W group and 13/96 (13.5%) in the standard group (*P* = 0.009, *Table 4*). The AT mechanism was assessed in all 16 participants and is presented in Supplementary material online, *Table S2*. Among six patients in the standard group undergoing redo ablation during AT, the most frequent mechanism of AT recurrence was a gap-dependent reentry involving the right-sided PVs (*n* = 3), followed by roof-dependent AT (*n* = 2) and typical flutter (*n* = 1).

## Discussion

### Main findings

The POWER PULSE study is the first RCT investigating vHPSD-70W using a flexible, enhanced-irrigation tip catheter in a purely paroxysmal AF cohort.

In this study comparing vHPSD (70 W/5–7 s) with standard ablation (30–40 W/20–30 s) we found that (i) vHPSD is non-superior to standard ablation for the primary efficacy endpoint of dormant PV conduction, (ii) vHPSD-70W is associated with a higher FPI rate of left-sided PVs and with significantly shorter procedure and RF duration, (iii) the incidence of SCLs is not significantly different between groups, (iv) freedom from any atrial arrhythmia at 12 months is similar between groups and (v) AT recurrence rate is significantly reduced with vHPSD-70W.

### Procedure efficacy

The primary efficacy endpoint, the number of reconnected PVs after adenosine testing, was not significantly different between groups despite a trend towards fewer reconnected PVs in the vHPSD-70W group. This confirms results of previous RCTs which did not find a difference in PV dormant conduction with HPSD.^15,17,18^ There was a trend towards a higher bilateral FPI with vHPSD-70W, which did not reach statistical significance. Nonetheless, FPI of left-sided PVs was significantly higher with vHPSD-70W in this study. First-pass isolation is an indicator of a high-quality lesion set and predictor of clinical ablation success.^3,21^ While the exact mechanism of improved FPI for left-sided PVI remains unknown, it may be related to an improved catheter stability with HPSD, as previously demonstrated.^5,22^ This secondary endpoint analysis should be interpreted with caution, since multiple comparisons increase the likelihood of type I error.

Dormant PV conduction was selected as primary endpoint in order to address the study hypothesis of superior lesion quality with vHPSD. While clinically more relevant rhythm outcomes are also determined by PVI durability, they are a less specific marker of lesion characteristics. Notably, performing additional ablation to eliminate dormant PV conduction improves rhythm outcomes^19^ and may therefore confound initial differences in acute lesion transmurality and irreversibility seen between the two groups.

In agreement with previous studies, vHPSD-70W was associated with a significantly shorter RF and procedure duration.^10–12,15,16^ Mean RF duration with vHPSD-70W (15.1 ± 6.7 min) was significantly shorter than with standard ablation (41.8 ± 18.3 min), which was similar to previously published trials.^19,23^ Procedure duration in the vHPSD-70W group (107.7 ± 34.2 min) was on average 24 min shorter than in the standard group and was comparable to the procedure duration reported for pulsed field ablation in the ADVENT trial (105.8 ± 29.4 min).^24^ Of note, the current procedure duration included 20 min of waiting time and the interval required for adenosine testing of each individual PV. Therefore, this study demonstrates a high procedure efficiency of vHPSD-70W, which is well comparable to other novel PVI technologies.

### Procedure safety

No significant difference in safety endpoints was found between vHPSD-70W and standard ablation. There was a trend towards a higher rate of steam pops with vHPSD. Nevertheless, no cardiac tamponade occurred and no statistically significant difference in symptomatic and silent thromboembolic events was found. The current study reported one TIA and a numerically lower incidence of SCL in the vHPSD arm. Silent cerebral lesions can serve as a surrogate parameter for procedure-related thromboembolic risk.^17,20,25^ The trend towards a lower SCL incidence with vHPSD (4.0% vs. 13.6%), is in line with a previous observational study demonstrating a significantly lower prevalence of silent stroke and a lower pro-thrombotic state with HPSD-50W.^25^ The latter study proposed that lower procedure and RF durations with vHPSD may not only be advantageous for increasing procedural efficiency, but may also reduce the risk of thromboembolism by less charring or microbubble formation.^25^

On the other hand, the SHORT-AF trial reported a numerically higher, but non-significant incidence of asymptomatic cerebral emboli with HPSD-50W (40% vs. 17%),^17^ and the POWER FAST III trial found a non-significant higher incidence of symptomatic embolic events with vHPSD-70W (2.8% vs. 0.0%).^18^ Importantly, both trials used different ablation catheters and had markedly longer procedure durations in the HPSD arm than the current trial. Furthermore, the pathophysiology of SCLs is multifactorial and not exclusively determined by RF energy application. Other factors such as patient characteristics, periprocedural anticoagulation, transseptal sheath management, procedure duration and the type of electroanatomic mapping system are associated with SCLs.^20,26–28^ While the risk of thromboembolic events with HPSD remains controversial, the current study suggests a similar safety profile between vHPSD-70W and conventional ablation, in agreement with a previous meta-analysis.^23^

### Clinical rhythm outcomes

Freedom from any atrial arrhythmia at 12-month follow-up was similar between groups. However, AT recurrences were significantly more common with standard ablation (10.4%) than with HPSD (1.0%) and required emergent hospitalization in 50% of cases. Atrial tachycardia recurrences occurring after first-time ablation of paroxysmal AF off antiarrhythmic drugs are likely due to non-durable ablation lesions favouring slow conduction zones and PV gap-dependent small-loop reentries.^29,30^ Indeed, our analysis of redo ablations revealed a gap-dependent reentry mechanism involving right-sided PVs in most AT cases in the standard group. This unexpected finding strongly suggests deficient lesion durability of standard ablation lesions, which may not be unmasked by intraprocedural measures such dormant PV conduction but rather becomes clinically evident during long-term follow-up.

### Impact of different HPSD settings

While the recently published POWER FAST III trial also investigated HPSD-70W,^18^ ablation catheters and RF duration (9 s/TactiCath™; 10 s/Thermocool Smarttouch® SF) differed from the current study. Catheter tip design is an essential determinant of biophysical characteristics. vHPSD requires enhanced catheter irrigation and accurate real-time temperature monitoring to avoid tissue overheating, steam pops and catheter charring. We therefore selected a flexible-tip catheter (FlexAbility™) due to its mesh-type irrigation kerfs and the very distal thermocouple, which had not been investigated in a HPSD-RCT so far.

Finding the optimal RF duration with vHPSD is also challenging due to its narrow safety-to-efficacy window.^5^ If duration is too short, this may compromise lesion durability, while long duration will diminish its safety. The current ablation protocol was based on previous data published by Bourier et al.^6^ FPI rate for 70W with a longer duration in POWER FAST III (71%) was considerably higher than the one reported in our study (43%).^18^ Nonetheless, our HPSD-70W/5–7 s protocol rendered a short mean procedure duration (108 min) and a 12-month freedom of any atrial arrhythmia of 76%. The optimal power and duration settings to achieve the right balance between efficacy and safety remain uncertain. An individualized application of vHPSD guided by local left atrial wall thickness has been recently proposed to enhance lesion transmurality while maintaining safety.^31^ A tailored RF application may therefore represent a promising approach, which requires validation in future studies.

The results of the current study support vHPSD as a promising technological innovation for reducing procedure duration, while maintaining procedural efficacy and a comparable safety profile.^4^

### Limitations

Despite the randomized-controlled design of the trial, sources of bias cannot be completely excluded. The fixed block size randomization bears the potential that the allocation of the last participant in each block is predictable. However, the study group was blinded to both the randomization method and the block size. The vHPSD-70W group showed a significantly higher diagnosis-to-ablation time, which may have impacted rhythm outcomes. This study was conducted with a non-contact force, flexible-tip catheter by operators experienced with vHPSD-70W. Therefore, the results may not be extrapolated to other HPSD protocols and ablation catheters, while reproducibility in other less experienced centres might be limited. The relatively low FPI rates might be attributable to the use of a non-contact force catheter and to possible map shifts which can occur with an impedance-based mapping system. Finally, while this study was designed to investigate efficacy outcomes, it was not powered for safety outcomes such as (clinically manifest) thromboembolic events and oesophageal fistula. Specifically, brain MRI was only performed in a subset of participants. Further large-scale studies are necessary for a definite appraisal of HPSD safety for specific ablation protocols and catheters.

## Conclusion

Very high-power short-duration with 70 W/5–7 s ablation for paroxysmal AF using a flexible tip catheter is non-superior to standard ablation in terms of PV dormant conduction and safety. A higher FPI rate of left-sided PVs and a shorter procedure and RF duration suggest an improved efficacy of vHPSD-70W. While freedom from any atrial arrhythmia is comparable to standard ablation, vHPSD-70W shows a significantly lower rate of atrial tachycardia recurrences.

## Supplementary Material

euaf105_Supplementary_Data

## Data Availability

The data underlying this article will be shared on reasonable request to the corresponding author.
