# Supplementary material for: Very high-power short-duration using 70W and a flexible tip ablation catheter for pulmonary vein isolation: the POWER PULSE randomized controlled trial
Source: Europace. 2025 Jul 21;27(8):euaf105. doi: 10.1093/europace/euaf105 (PMC12365637; doi:10.1093/europace/euaf105)
Supplement: euaf105_Supplementary_Data [file euaf105_supplementary_data.docx]

**Supplementary Material**

1. **Inclusion and exclusion criteria**

**Inclusion criteria**

- Patients aged >18 years and <80 years with symptomatic, documented paroxysmal atrial fibrillation
- At least one unsuccessful medical therapy with a beta-blocker and/or class I or III antiarrhythmic drug
- Effective oral anticoagulation with a vitamin-K antagonist or with a direct oral anticoagulant established for at least 4 weeks prior to ablation
- Antiarrhythmic drugs stopped ≥3 half-lives prior to ablation

**Exclusion criteria**

- Persistent atrial fibrillation
- Prior left atrial catheter ablation
- Reversible causes of atrial fibrillation (e.g. hyperthyroidism or severe electrolyte disturbance)
- Heart failure with ejection fraction <35%
- Catheter ablation, coronary intervention or heart surgery within 3 months prior to ablation
- Left atrial thrombus
- Contraindications for oral anticoagulants
- Contraindication for adenosine challenge (e.g. asthma bronchiale)
- Prior esophageal or gastric surgery

1. **Study endpoints**

**Primary endpoint**

- Number of reconnected pulmonary veins after 20 minutes of waiting time and adenosine challenge

**Secondary endpoints**

- Recurrence of any atrial arrhythmia at 12 months after a blanking period of 6 weeks
- Type of atrial arrythmia recurrence
- Procedure duration, radiofrequency duration, fluoroscopy time
- First-pass isolation of ipsilateral pulmonary veins (not requiring additional ablation at the carina)
- Incidence of steam pops
- Procedure- and ablation-related complications: groin complications, cardiac tamponade, pulmonary vein stenosis, transient ischaemic attack/stroke, symptomatic thermal esophageal lesions, cardiac arrest, death
- Incidence of silent cerebral lesions on brain magnetic resonance imaging
- Incidence of redo ablations
- Chronic pulmonary vein reconnection on redo ablation

**Suppl. Table 1: Redo ablations**

|  | **vHPSD-70W**  **(n = 25)** | **Standard ablation (n = 30)** | **p value** |
| --- | --- | --- | --- |
| Any PV reconnection, n (%) | 23 (92.0) | 28 (93.3) | 1.000 |
| Mean reconnected PVs, n (%) | 2.6 ± 1.3 | 2.5 ± 1.2 | 0.858 |
| Redo PVI of both PV pairs, n (%) | 19 (76.0) | 22 (73.3) | 0.821 |
| Redo PVI of right-sided PVs, n (%) | 21 (84.0) | 27 (90.0) | 0.689 |
| Redo PVI of left-sided PVs, n (%) | 21 (84.0) | 23 (76.7) | 0.498 |
| Lines, n (%) | 3 (12.0) | 8 (26.7) | 0.176 |
| CTI, n (%) | 2 (8.0) | 6 (20.0) | 0.269 |
| CTI *de novo*, n (%) | 1 (4.0) | 4 (13.3) | 0.362 |
| Roof, n (%) | 1 (4.0) | 2 (6.7) | 1.000 |
| anterior, n (%) | 1 (4.0) | 0 (0.0) | 0.455 |
| Other, n (%) | 5 (20.0) | 4 (13.3) | 0.717 |
| SVC isolation, n (%) | 1 (4.0) | 1 (3.3) | 1.000 |
| slow pathway ablation, n (%) | 1 (4.0) | 2 (6.7) | 1.000 |
| PVC ablation, n (%) | 1 (4.0) | 0 (0.0) | 0.455 |
| ablation of fractionated potentials, n (%) | 1 (4.0) | 1 (3.3) | 1.000 |
| ablation of focal atrial tachycardia in the NCC, n (%) | 1 (4.0) | 0 (0.0) | 0.455 |

CTI=cavotricuspid isthmus, NCC=non-coronary cusp, PV=pulmonary vein, PVC=premature ventricular complexes, PVI=pulmonary vein isolation, SVC=superior vena cava

**Suppl. Table 2: Atrial tachycardia recurrences on extended follow-up**

| **No.** | **Participant number** | **Recurrence (days)** | **Group** | **Emergent re-admission due to AT** | **Redo ablation** | **Rhythm on redo ablation** | **Mechanism of reentry** |
| --- | --- | --- | --- | --- | --- | --- | --- |
| 1 | 39 | 108 | Standard | yes | yes | SR | Likely PV-dependent (all PVs reconnected) |
| 2 | 50 | 429 | vHPSD-70W | no | no | / | unknown |
| 3 | 71 | 947 | Standard | no | yes | SR | Likely typical flutter after prior CTI ablation (ECG-documented); all PVs reconnected |
| 4 | 99 | 1265 | Standard | no | no | / | unknown |
| 5 | 110 | 108 | Standard | no | yes | SR | Likely PV-dependent (all PVs reconnected) |
| 6 | 114 | 593 | Standard | yes | no | / | unknown |
| 7 | 119 | 230 | Standard | yes | no | / | unknown |
| 8 | 128 | 42 | Standard | no | yes | AT | Typical flutter (CL 205 ms), reconnection of right-sided PVs |
| 9 | 129 | 103 | Standard | no | yes | SR | Roof-dependent (CL 340 ms, induced on burst-stimulation), reconnection LSPV + RIPV |
| 10 | 132 | 230 | Standard | no | yes | AT | Right-sided PVs (CL 250 ms), gap-dependent |
| 11 | 136 | 180 | Standard | yes | yes | AT | Roof-dependent (CL 260 ms), LIPV reconnection |
| 12 | 156 | 92 | Standard | yes | yes | AT | Right-sided PVs (CL 240 ms), gap-dependent |
| 13 | 162 | 211 | vHPSD-70W | no | yes | AT | Roof-dependent (CL 260 ms) with slow-conduction zone at the anterior wall in a pre-existing low-voltage area (not PV-dependent) |
| 14 | 173 | 99 | Standard | yes | no | / | unknown |
| 15 | 175 | 394 | vHPSD-70W | no | yes | SR | Likely typical flutter (ECG-documented), reconnection of right-sided PVs and LIPV |
| 16 | 200 | 184 | Standard | no | yes | AT | Right-sided PVs (CL 290 ms), gap-dependent |

AT=atrial tachycardia, CL=cycle length, CTI=cavotricuspid isthmus, PV=pulmonary vein, LIPV=left inferior pulmonary vein, LSPV=left superior pulmonary vein, RIPV=right inferior pulmonary vein, SR=sinus rhythm
